# Sport-related Concussion Characteristics and Time-loss Among Male and Female Community Rugby Union Players Across 6 Seasons

**DOI:** 10.1177/19417381261474860

**Published:** 2026-10-07

**Authors:** Laura C. Power, Tom M. Comyns, Helen Purtill, John J.E. Mulvihill, Kilian L. Bibby, Caithríona Yeomans, Patrick Dolan, Michael W. Collins, Anthony P. Kontos, Ian C. Kenny

**Affiliations:** Department of Physical Education and Sport Sciences, University of Limerick, County Limerick, Ireland, Health Research Institute, University of Limerick, County Limerick, Ireland, and Sport and Human Performance Research Centre, University of Limerick, County Limerick, Ireland; Department of Physical Education and Sport Sciences, University of Limerick, County Limerick, Ireland, Health Research Institute, University of Limerick, County Limerick, Ireland, and Sport and Human Performance Research Centre, University of Limerick, County Limerick, Ireland; Health Research Institute, University of Limerick, County Limerick, Ireland, and Department of Mathematics and Statistics, University of Limerick, County Limerick; Health Research Institute, University of Limerick, County Limerick, Ireland, Sport and Human Performance Research Centre, University of Limerick, County Limerick, Ireland, and School of Engineering and Bernal Institute University of Limerick, County Limerick, Ireland; Department of Physical Education and Sport Sciences, University of Limerick, County Limerick, Ireland, Health Research Institute, University of Limerick, County Limerick, Ireland, and Sport and Human Performance Research Centre, University of Limerick, County Limerick, Ireland; Medical Department, Irish Rugby Football Union, Dublin, Ireland; Department of Physical Education and Sport Sciences, University of Limerick, County Limerick, Ireland, Health Research Institute, University of Limerick, County Limerick, Ireland, and Sport and Human Performance Research Centre, University of Limerick, Limerick, County Limerick, Ireland; Department of Orthopaedic Surgery, University of Pittsburgh School of Medicine, University of Pittsburgh Medical Center Sports Medicine Concussion Program, Pittsburgh, PA, USA; Department of Orthopaedic Surgery, University of Pittsburgh School of Medicine, University of Pittsburgh Medical Center Sports Medicine Concussion Program, Pittsburgh, PA, USA; Department of Physical Education and Sport Sciences, University of Limerick, County Limerick, Ireland, Health Research Institute, University of Limerick, Limerick, County Limerick, Ireland, Sport and Human Performance Research Centre, University of Limerick, County Limerick, Ireland, and Lero, the Research Ireland Centre for Software

**Keywords:** concussion recovery, mild traumatic brain injury, mTBI, return-to-play, rugby injury, rugby science

## Abstract

**Background:**

Sport-related concussion (SRC) is a common injury in Rugby Union. There is currently a paucity of prospective research in SRC specific data in both male and female community Rugby Union players, which may restrict SRC prevention, recognition, and management practices.

**Hypothesis:**

Adult community SRC injuries would be common in match-play, forward players, and in the tackle event, alongside increased time-loss following SRC in female players.

**Study Design:**

Prospective cohort study.

**Level of Evidence:**

Level 2.

**Methods:**

Data were collected across 6 seasons between 2017/18 and 2023/24, from 5740 male and female player-seasons registered with the Irish Rugby Injury Surveillance system. SRC injury data included player demographics, setting (match, training), mechanism of injury, timing occurrence, and time-loss.

**Results:**

Of the 456 reported SRCs, 93.9% occurred on match days and 6.1% during training. SRCs reported during matches were more likely to occur during the second half (males 63.5% [95% CI 58.5%, 68.4%]; females 72.1% [95% CI 58.7%, 85.5%]). For male players, most match-day SRCs occurred when tackling 55.9% (95% CI 50.9%, 60.8%), compared to more evenly distributed mechanisms observed in females (tackling 38.6%; being tackled 29.5%; ruck 18.2%). Severe SRC time-loss injuries (>28 days absence) were significantly more likely in female players compared with male players (46% vs 30%; *P* = 0.013). Annual SRC prevalence ranged from 4.0% to 9.7% for male players and 3.1% to 7.0% for female players across the 6 seasons.

**Conclusion:**

In adult community Rugby Union most recorded SRCs occurred during the tackle event, match-play, and were more likely to occur in the second half. Female players may be more at risk for greater time-loss following SRC than male players.

**Clinical Relevance:**

Sports medicine staff, coaches, and players should be aware of match time and mechanism of injury (eg, tackling) when SRCs are more likely to occur, alongside the potential increased time-loss risk in female players, to support SRC recognition and recovery.

Rugby Union (hereafter rugby) is a physically demanding game, with both low-intensity (eg, jogging and walking) and high-intensity (eg, sprinting, tackling, and mauling) components.^5,22,23,29,38^ Existing literature indicates that injury frequently occurs as a result of the high-intensity components of the game, such as the tackle event.^12,22,28,36,38^ In particular, there are high rates of sport-related concussion (SRC), in rugby, in both the community and professional game.^10^ An SRC is defined as “a traumatic brain injury caused by a direct blow to the head, neck, or body resulting in an impulsive force being transmitted to the brain that occurs in sports and exercise-related activities”.^26^ The heterogeneity of SRC characteristics^19^ across different populations adds a layer of complexity to SRC research. For example, females have been found to have prolonged SRC recovery duration.^16^

Rugby-specific SRC risk factors to-date have been previously identified as playing position^4^ and level of play.^31^ However, the preponderance of SRC-specific data are focused on the elite game,^10,27,35^ despite the majority of rugby players participating at a nonelite level, and high levels of SRC prevalence rates being found at a community level.^32^ Research at the elite playing level may disregard the differences in access to medical care,^2^ player demographics,^30^ and likely coaching skills. In addition, community SRC literature to-date frequently focuses on male players,^22,28^ match-day injuries,^28^ or is published as part of whole-body injury surveillance analysis^17,38^ which may frequently lack detailed elements of SRC injuries when reported within a broader dataset. Moreover, it is difficult to discern currently the accuracy of practical implications of community-level rugby SRC literature, due to a mismatch in the growth of rugby participation^37^ and paucity of publications of prospective SRC-specific data.

The aim of this study is to characterize SRCs in male and female community rugby players, in relation to prevalence of injury, playing position, mechanism of injury, match timing of injury, and time-loss. We hypothesize that SRCs would more commonly be reported in match-play than training, during tackle events than other mechanisms, and forward players than backs in both sexes, alongside female sex association with greater time-loss from SRC.

## Methods

### Study Design and Participants

This research involved a prospective cohort study of adult male and female community rugby players, from July 2017 to May 2024 (6 seasons). In Ireland, there are 90,029 registered players within Irish Rugby Football Union (IRFU) clubs,^15^ with the highest level of community rugby being the All-Ireland League (AIL), comprising currently of 60 clubs (50 male; 10 female). Over 6 seasons, on average, 20 male clubs and 5 female clubs (967 players) annually, or overall, 5740 player-seasons (where 1 player participating in 1 season is 1 player-season) participated in the Irish Rugby Injury Surveillance (IRIS) project which was approved by University of Limerick’s Faculty of Education and Health Sciences research ethics committee (2016_06_19_EHS), in accordance with the Declaration of Helsinki.

### Procedures and Data Collection

All injuries were documented by a designated and trained injury recorder at each club during both match and training settings,^39^ however solely SRC injuries were investigated for the purpose of this study. All injury recorders received training in system use prior to data collection.^39^ Injury data were collected as per international consensus,^9^ meaning that any injury that resulted in a 24-hour time-loss was recorded as a time-loss injury. The definition of SRC, along with the injury severity (time-loss) grouping system have been described previously.^9^ Throughout each season, a weekly data audit was conducted by a member of the IRIS research team to ensure data accuracy.^39^ If a player suffered multiple SRCs, the first recorded injury was used for the purpose of the player-level demographic and prevalence analyses, while all subsequent SRCs were analyzed to establish a profile of all concussion injuries. Any data collection for the 2020/21 season was excluded from analysis as this season was incomplete and nonrepresentative, due to the disruption from the COVID-19 pandemic.

Each player self-reported demographic information at the start of data collection including date of birth, sex, height, body mass, and playing position. Recorded injury characteristics included the type and mechanism of injury, timing occurrence (eg, first quarter of game and training set plays), date of injury, date of return-to-play, time-loss (measured in days), and setting (match vs training). SRC injuries were categorized into new (first-recorded SRC injury) or recurrent (all subsequent SRC injuries). Data were collected on a secure, web-based injury surveillance system, IRISweb, which has been previously documented in the literature,^38,39^ and reported an average compliance rate of 89.2% (±5.0).

### Statistical Analyses

Summary statistics are presented as a number (percentage) for categorical data and median with interquartile range (IQR) for numeric data. Annual SRC prevalence was calculated for each season as the percentage of registered players who reported at least 1 SRC during a season, with an associated 95% confidence interval. It should be noted that the 2023/24 season was in line with the first year of a 2-year trial related to the lowering tackle height rule changes, where the legal tackle height was adjusted from the armpit (axilla) to below the base of the sternum (ie, a belly tackle), enforced nationally by referees.^14^ This was a 2-season trial to reduce the risk of head impacts during the tackle event,^14^ which may have affected the prevalence analyses, but is outside of the remit of the study.

SRC time-loss was categorized in accordance with rugby-specific literature,^9^ as up to 28 days (moderate) and >28 days (severe), for all SRC injuries. It is worth noting that the minimum return to play timeframe for adult community players in Ireland is 21 days,^13^ so the category of “moderate” time-loss injuries are generally between 21 and 28 days. The Chi-square test of independence was used to examine between group differences for categorical data. A 5% level of statistical significance was used throughout analyses. IBM SPSS Statistics (Version 29.0.1.0 (171)) was used to perform all statistical analyses.

## Results

### Player-level Analysis

There were 369 players who reported an SRC injury during the 6 seasons (n = 321 male; n = 48 female), demographics and playing position are presented in Table 1. Forwards were more likely to suffer SRCs (61.5% male; 54.2% female) than backs (38.5% male; 45.8% female).

**Table 1.** Player demographics (n = 369)

	Male (n = 321)		Female (n = 48)	
	n	Median (IQR)	n	Median (IQR)
Age (years)	275	23.8 (5.5)	40	25.4 (7.4)
Height (cm)	305	183.0 (9.0)	47	173 (10.0)
Mass (kg)	307	95.0 (18.0)	47	76.0 (20.0)
Player position		n (%)		n (%)
Forward		192 (61.5%)		26 (54.2%)
Back		120 (38.5%)		22 (45.8%)

IQR, interquartile range.

### SRC Prevalence

Annual SRC prevalence for male and female players is presented in Figures 1(a) and 1(b), respectively, by season of play. Average annual SRC prevalence from the 6 seasons was 6.68% (95% CI 5.97%, 7.38%) for male players and 5.14% (95% CI 3.73%, 6.56%) for female players.

**Figure 1.**
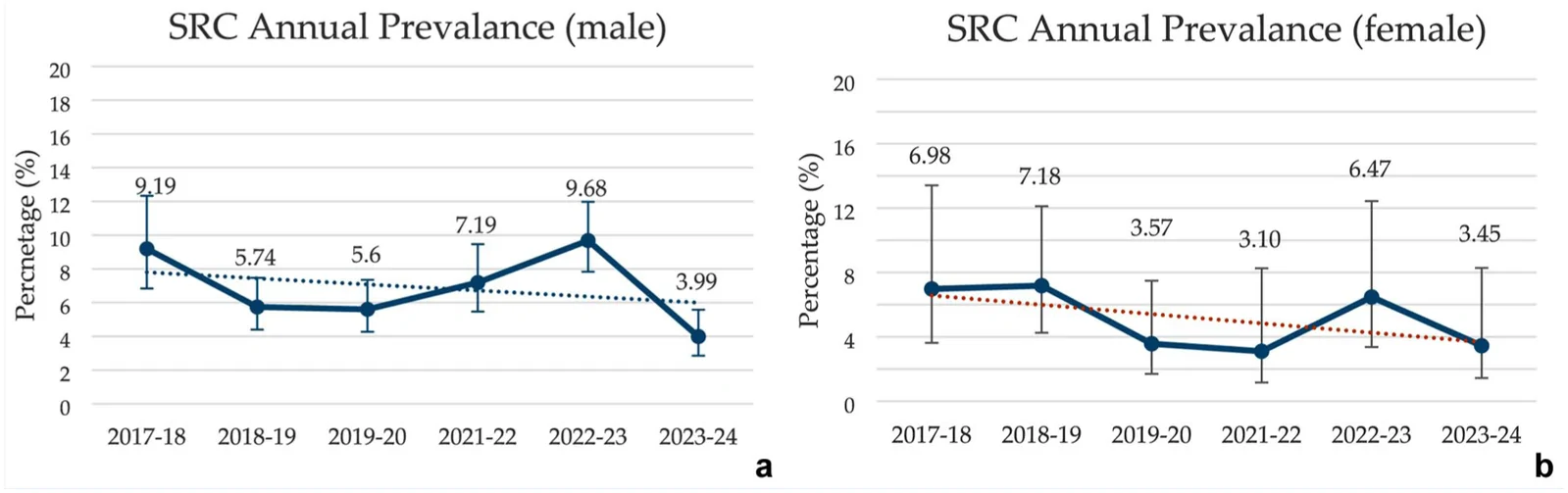
(a) SRC male prevalence across seasons. (b) SRC female prevalence across seasons.

### SRC Characteristics

There were 456 total SRCs recorded across 6 seasons, from 369 unique players, 73 of which sustained at least 1 repeat SRC. Of the 456 reported SRCs, 93.6% occurred in matches and 6.1% during trainings. Characteristics of reported match SRCs are summarized in Table 2. The majority of match injuries included new SRCs (78.9% males; 90.9% females), compared to recurrent SRCs (21.1% males; 9.1% females). The tackle event (tackling and being tackled) accounted for 77.6% of all male SRCs and 68.1% of all female SRCs, followed by the ruck event (12.3% male; 18.2% female). SRCs reported during matches were more likely to occur during the second half (combination of third and fourth quarter [40-80 minutes]; males 63.5% [95% CI 58.5%, 68.4%], females 72.1% [95% CI 58.7%, 85.5%]).

**Table 2.** Descriptive findings of rugby specific variables for all match-day SRCs

		Match-day SRC injuries	
		**Male SRC** (n = 383)	**Female SRC** (n = 44)
		n (%)	n (%)
New/recurrent injury	New	302 (78.9)	40 (90.9)
	Recurrent	81 (21.1)	4 (9.1)
Mechanism	Tackling	214 (55.9)	17 (38.6)
	Being tackled	83 (21.7)	13 (29.5)
	Ruck	47 (12.3)	8 (18.2)
	Other*^a^*	39 (10.2)	6 (16)
Match occurrence	First quarter (0-20 minutes)	44 (12)	3 (7.0)
	Second quarter (20-40 minutes)	90 (24.5)	9 (20.9)
	Third quarter (40-60 minutes)	111 (30.3)	15 (34.9)
	Fourth quarter (60-80 minutes)	122 (33.2)	16 (37.2)
	Other*^b^*	2 (0.5)	8 (2.1)

a Includes other mechanisms that were logged in low volume such as scrum, line-out, accidental collision, and aerial duel.

b Includes other occurrences such as postmatch.

Training SRC injuries accounted for 21 (5.2%) of male SRCs and 7 (13.5%) of all female SRCs. Contact drills were the most reported mechanism for both male (71.4%; n = 15) and female (42.9%; n = 3) training SRC injuries. The tackle event specifically was responsible for most training SRCs for males (52.4%; n = 11) and females (57.2%; n = 4).

### SRC Time-loss

SRC time-loss data were available from 454/456 (99.6%) of all recorded SRC injuries. Career-ending or retirement following SRC injury occurred for 7 players (1.5% of SRC injuries). Male SRC median (IQR) time-loss was 24 (21-33) days. Female SRC time-loss was 28 (21-42) days.

SRCs in female players were more likely to result in time-loss >28 days compared with SRCs in males (46.2% vs 30.4%; *P* = 0.022), as can be seen in Figure 2.

**Figure 2.**
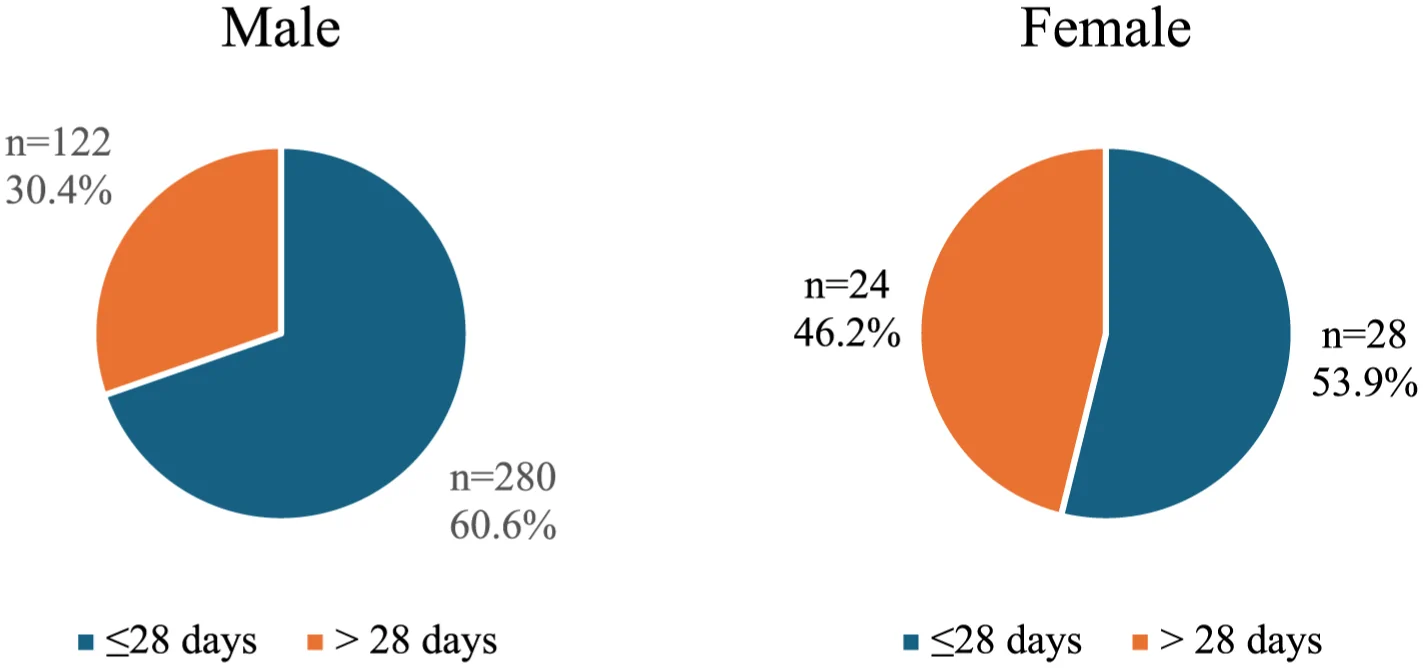
Male and female SRC time-loss (day’s absence).

## Discussion

This study characterized long-term trends in SRCs in male and female rugby players across 6 seasons of matches and training. Findings showed higher frequencies of SRCs in match-play (specifically the third and fourth quarter), tackling events, and the forward position, for both male and female players. Furthermore, this study identified a high occurrence of >28 days time-loss following SRC, by 30% of male and 46% of female players. SRC prevalence showed fluctuations throughout the seasons for both sexes, however the fluctuations were less prominent than prior prospective SRC research in professional males (4%-20%).^35^ The fluctuations in prevalence, particularly in the male match-day SRC injuries, seen in the 2023/24 season may have been affected by the lowering of tackle height trial’s rule changes.^14^

### SRC Characteristics

Forwards sustained more SRC injuries than backs in both male and female cohorts, however the gap was larger in male (61.5% forwards) than female (54.2% forwards) players. These findings align with previous research in that the forward position has a heightened risk for head injury.^33^ It is unclear whether this increase in forward SRCs is due to intrinsic risk factors, or potentially due to different relative exposure from their counterparts, with 8 forwards and 7 backs on pitch during play.^36^ Little is known regarding sex differences in position and injury risk. Lack of comparability between male and female sporting contexts has been highlighted previously in the literature.^25^ Thus, position specific risk factors in future research should be investigated with consideration of potential sex-specific influences.

Rugby-specific findings for SRCs reflect previous work which denotes the tackle event as being the specific contact mechanism for SRC injury for both sexes.^22,28,36,38^ Previous research have shown a player’s tackle action and technical ability to be risk factors for injury in rugby,^3^ such as, but not limited to, the initial impact location on the ball-carrier, or players diving for the ball.^12,24^ This may indicate that a focus from stakeholders on improving the tackle technique may act as an SRC preventive measure.^24^ Variables seen in female rugby such as delayed starting age (a lower training age) and differences in coaching activities such as tackling or being tackled^6^ may be considerable factors for the increase in female ball-carrier SRC injuries, in comparison to their male counterparts.

This study produces novel findings of contact drills within training being the primary occurrence of male and female training SRCs in a community rugby cohort. Limiting contact in training has been found to have a 64% reduction in training SRC injuries in other codes,^7^ which rugby stakeholders should be aware of as a potential risk-aversion strategy within a training environment. However, it is currently unknown whether reduction in training contact outweighs the potential benefits of tackle technique work. Future research is needed to determine optimal and tailored SRC prevention strategies in training, in line with the player and/or teams’ hierarchy of priorities.

Both male and female SRCs were predominantly accounted for by match injuries, particularly in latter stages of play, similar to previous SRC research.^10,11,28^ This may be due to match variables such as lack of concentration, fatigue, or accumulation of match stress later in play, particularly for community players who may be less conditioned than elite-level athletes.^8,36^ Training variables such as lower intensity or the presence of medical supervision^10^ may be contributing factors for the low levels of training SRCs reported.

### Time-loss from SRC

In terms of SRC time-loss, current research suggested that 10% to 15% of players will suffer from prolonged SRC recovery (over 1 month).^1,21,26^ In comparison, this study unveils 3 to 5 higher rates of prolonged SRC for female and male community rugby players, respectively, the cause of which remains unclear. As a result of the IRFU’s 21-day return-to-play policy, the severity may be influenced by the player’s eligibility for unrestricted play following the minimum graduated return to play protocol. There are multiple alternative potential rationale for this prolonged time-loss, such as initial symptom severity, lifetime concussion history, and access to healthcare professionals,^16,20,34^ however current literature lacks clarity of these in community rugby-specific contexts.

These findings support previous research that females may suffer from increased SRC time-loss than males,^18^ where females appear to be at a higher risk of taking >28 days to recover than males. This may be due to a range of possible factors such as heightened symptom reporting in female athletes.^16^ This further supports the requirement to analyze sex-specific SRC data separately in research and implement sex-specific recovery trajectories into clinical decision making, when managing recovery expectations within the sporting population.

### Limitations

The current study was limited by several factors, including the infrequent use of nonmedical professionals as injury recorders at some clubs. In addition, some clubs did not have a medic present or had different medics across sessions (training/match) or across different clubs. However, 2-step injury reporting (time of injury reporting and time of injury clearance) processes are in place to mitigate this potential issue. Prevalence was computed from the list of registered players on the IRIS system. Therefore, true prevalence rates for SRC may actually be higher than reported here. Furthermore, there are a smaller number of female clubs participating in the study, coupled with large turnover, which may affect findings. Lastly, within the study’s data collection period, 1 season included data during which a trial law change of a lower tackle height was in progress, which may have influenced SRC prevalence during that year. This trial, which continued 1 year beyond the study time period, will be the focus of future research on the effect of this law change on SRC-related outcomes.

## Conclusion

SRC prevalence in Rugby Union fluctuated for both males and females across the study time period. SRC injuries were most common for players in the forward position, matches compared with training, tackling compared with other contact and noncontact mechanisms, and the third and fourth quarter of match-play. Nearly one-third of males and almost one-half of females experienced >28 days time-loss following SRC. SRCs in female players were more likely to result in time loss greater than 28 days compared with male players. Sports medicine staff, coaches, and players should be aware of factors associated with increased risk for SRC (eg, latter stages of match play) and time loss (eg, female sex) from this injury. Future research should focus on highlighting potential pre- (eg, SRC history) and postinjury (eg, continued play) variables within this cohort which may be associated with SRC and time-loss from injury, to better inform future clinical decision making and SRC management in rugby.
